# Unbalanced international collaboration affects adversely the usefulness of countries’ scientific output as well as their technological and social impact

**DOI:** 10.1007/s11192-016-2126-8

**Published:** 2016-09-17

**Authors:** Sonia R. Zanotto, Cristina Haeffner, Jorge A. Guimarães

**Affiliations:** 1 Programa de Pós-Graduação em Educação em Ciências: Química da Vida e Saúde (PPGQVS), Universidade Federal do Rio Grande do Sul - UFRGS and Instituto Brasileiro de Geografia e Estatística (IBGE - RS), Porto Alegre, RS Brazil; 2 Biblioteca do Instituto de Educação e Pesquisa (IEP), Hospital Moinhos de Vento, Porto Alegre, RS Brazil; 3 Hospital de Clínicas de Porto Alegre and Centro de Biotecnologia, Universidade Federal do Rio Grande do Sul - UFRGS, Av. Bento Gonçalves, 9500, C.P. 15005, CEP 91501-970 Porto Alegre, RS Brazil

**Keywords:** Scientific production, Impact, International collaboration, Double-counting of articles, Bibliometric analysis

## Abstract

The unbalanced international scientific collaboration as cause of misleading information on the country’s contribution to the scientific world output was analyzed. ESI Data Base (Thomson Reuters’ InCites), covering the scientific production of 217 active countries in the period 2010–2014 was used. International collaboration implicates in a high percentage (33.1 %) of double-counted world articles, thus impacting qualitative data as citations, impact and impact relative to word. The countries were divided into three groups, according to their individual contribution to the world publications: Group I (24 countries, at least 1 %) representing 83.9 % of the total double-counted world articles. Group II (40 countries, 0.1–0.99 % each). Group III, 153 countries (70.5 %) with <0.1 % and altogether 1.9 % of the world. Qualitative characteristics of each group were also analyzed: percentage of the country’s GNP applied in R&D, proportion of Scientists and Engineers per million inhabitants and Human Development Index. Average international collaboration were: Group I, 43.0 %; Group II, 55.8 % and Group III, 85.2 %. We concluded that very high and unbalanced international collaboration, as presented by many countries, misrepresent the importance of their scientific production, technological and social outputs. Furthermore, it jeopardizes qualitative outputs of the countries themselves, artificially increasing their scientific impact, affecting all fields and therefore, the whole world. The data confirm that when dealing with the qualitative contribution of countries, it is necessary to take in consideration the level of international cooperation because, as seen here, it can and in fact it does create false impression of the real contribution of countries.

## Introduction

Today, most countries, no matter how developed they are, produce scientific articles in several areas and specific fields of knowledge. In fact, as observed by the Australian Academy of Sciences (2010), “Developing countries have increased their participation in science and technology research” and thus “scientists from more developed countries are building personal partnerships with these countries”. Presently, a total of 251 specialized fields are listed in the Web of Science (Thomson Reuters’ InCites Data Base), and a total of 217 countries are publishing scientific articles. According to the ESI Data Base, the 251 fields are summarized in 22 great areas of knowledge. Although the number of countries publishing articles in each specific field can vary largely, very few among the 22 areas have less than one hundred countries contributing with research and publications. On the other hand, the great majority of scientific articles are published by a small group of countries most occupying the highest level of economic welfare and social development, thus corroborating the notion that “research and innovation contribute in the short and long terms to prosperity and competitiveness, as well as to the resolution of society’s greatest challenges in areas like health, energy and security” (Wadworth 2014). In this context it is important to consider the thought of William Press (Press 2013) about the social impact of science in the USA: “what is so special about science (and how much we should spend on it)?”

Scientific international collaboration, as measured by co-authorship and publications has been growing constantly and the percentage of co-authored articles has more than doubled in the last two decades (Wagner et al. 2015). These authors observed that the open possibility of co-authorship has attracted productive scientists to participate in international projects. Nevertheless, they emphasized that “National governments could gain efficiencies and influence by developing policies and strategies designed to maximize network benefits—a model different from those designed for national systems”. It has been also emphasized that international co-authored publications receive greater number of citations (Glänzel and Schubert 2001; Persson et al. 2004). This distinctive pattern also occurs in more developed countries like the Europeans ones (Narin et al. 1991). Such considerations are important issues for the analysis presented in this work.

Since we studied the proportion of international collaboration pattern as a whole, our findings could not contradict the notion that “the more basic the field, the greater the proportion of international co-authorships” (Frame and Carpenter 1979; Luukkonen et al. 1992). Also, we did not distinguish between collaboration and co-authorship as emphasized by Katz and Martin (1997). Actually, as mentioned by Subramanyam (1983), “the assessment of collaboration using co-authorship is by no means perfect, it nevertheless has certain advantages: it is invariant and verifiable; given access to the same data-set, other investigators should be able to reproduce the results”. Several authors have shown that international collaboration increases visibility of the scientific work (Katz and Martin 1997) and the resultant publications receive higher consideration (Van Raan 1988) or, in other words, publications resulting from international collaboration produce “more positive effects on the quality of the output when compared to research without collaboration” (Abramo et al. 2009). Other authors made similar observations (Meneghini 2010; Tahamtan et al. 2016; Meneghini et al. 2008; Akre et al. 2011; Leite et al. 2011). Concerning to this, Smith et al. (2014) studying journal placement and citation performance of articles, observed that “the relative success of articles can be holistically assessed, yielding new insights into the scientific impact of individual countries and cross-national collaboration”. As a consequence, it can be concluded that international collaboration produces a number of benefits for participating researchers “that ultimately translate into greater scientific visibility, quality and impact”. In fact this has been shown by several authors (Van Raan 1988; Martin-Sempere et al. 2002; Barjack and Robinson 2007; Bozeman and Corley 2004) allowing to conclude that “international collaboration produces real and remarkable results in the scientific performance of research groups” (Abramo et al. 2009; Gevers 2014). Actually, the subject of cooperation can be considered an international enterprise (Van Raan 1997). This is especially true when it includes highly productive scientists from the most developed countries. In such cases there is a great chance that articles will be published in a journal with a higher impact factor, thus resulting in significant increase in citations and thus of the impact of the publication (Katz and Martin 1997; Abramo et al. 2009; Smith et al. 2014). This phenomenon has to do with the known effect of authors’ affiliation (Akre et al. 2011). On the other hand for the less developed countries international collaboration is based in the concept assumed by funding agencies and policy makers that it is “a good thing” that “should be universally encouraged” (Katz and Martin 1997). Furthermore, it is well known that the number of published articles in a specific field depends on the size of the scientific community of that area or field (Gaffriau and Larsen 2005). This has been shown by Smith et al. (2014), who analyzed eight different disciplines in relation to two categories of indicators: journal placement and citation performance. They found that those disciplines “with more countries in their affiliations performed better in both categories”. In other words, the size of the scientific production is related to the sum of all countries’ researchers working on a particular area or field which represents the total world production in that field, as observed by Bornmann et al. (2012). On the other hand, other indicators, such as individual counting of articles, total citations, the impact and international cooperation level, as well as other qualitative science-derived issues are dependent of other unrelated factors. Of particular interests in this aspect is the proportion of collaborative articles resulting from co-authorship among researchers from different countries, especially in the case of countries with a small number of published articles, which reflects in an insignificant quantitative and, even less, qualitative contribution to the world scientific output.

Concerning the contribution of the 217 countries to the world output, one can find that by counting the number of documents country by country there is an 1.33-fold increase in the number of counted articles as compared to the amount of documents effectively published by the whole world. The difference accounts for the double-counting effect due to publications produced in co-authorship by authors from two or more countries. In such cases, documents produced by each country in collaboration with other countries will be counted at least twice. As pointed before (Beys-da-Silva et al. 2014) the double-counting trait is derived from the fact that whenever a publication includes cooperation between or among researchers linked to two or more institutions, countries or fields of knowledge, these articles will be counted more than once (Almeida and Guimarães 2013). Double-counting is inherent to the bibliometric measures of scientific production of the countries, research fields and institutions. In our study we identify it as a trait related to high number of articles resulting from international collaboration, especially from the less productive countries with the more developed ones.

Here we show that international collaboration may have an unconstructive influence affecting directly the usefulness of the scientific impact when such collaboration involves countries with very small number or articles and thus a low scientific output. Actually, a great number of countries still present a very low scientific out put, and the double-counting feature “masks” the real importance of their genuine contribution to the advance of the world science and may hinder the development of each of these countries. Thus, studies ignoring the factors affecting bibliometric indicators, such as unbalanced scientific collaboration and contribution of double-counting to high impact, often induce a biased analysis of the relevance to the world of countries with lower scientific output. In fact this is a common fault that must be avoided when such scientometric analyses are made. Thus, it is important to know the dimensions of these distortions because they can and in fact, they do introduce bias in the bibliometric analysis of science output, especially when comparing countries and subject areas of research. In this work we compare the data of both indicators, that is to say, with and without considering double-counting.

An expected benefit of international collaboration among countries could be attributed to the expectation of knowledge transfer (Katz and Martin 1997), actually as mention by the authors “Collaboration is one way of transferring new knowledge, especially tacit knowledge”. However as shown here, this seems not to be occurring in the cases of unbalanced collaboration as identified in our study. Although several studies dealing with international collaboration have been published little attention has been given to the deleterious effect of excessive co-authorships among researchers from the most developed countries and that of authors from undeveloped countries.

In the present work we aimed to explore the deleterious influence of unbalanced levels of international collaboration to the world’s scientific indicators. Such problem expressed as double-counting of articles, involves a large number of countries with very little proper and autonomous scientific production. The observed situation creates an illusory level of scientific performance, which resulted from the unbalanced collaboration between authors from these countries with the authors from more productive ones. Another aspect concerning science development is how it relates to long term prosperity, welfare and competitiveness. This study also shows some relationships implicating these indicators.

## Methodology

In this work we used the qualitative and quantitative bibliometric indicators with the purpose of evaluation of the contribution of the countries in the world scientific production. The InCites’ Data Base (Thomson Reuters, Philadelphia, USA, available online through the Portal of Periodicals offered by Fundação Coordenação de Aperfeiçoamento de Pessoal de Nível Superior, CAPES, Brazil) was used as the source of scientific data. The InCites’ Data Base provides scientific information of countries, institutions, and individuals as well as to the various areas of knowledge. This extensive Data Base allows its use for both quantitative and qualitative assessment of information covering all scientific areas, including the humanities and social sciences. Here the data of publications in referred journals, including: Number of published documents, Percentage of World Articles, Citations, Impact, International Collaboration Index (ICI) and the Impact Relative to World (IRW), were extracted. Data analysis allowed the acquirement of each country’s productivity index, enabling comparisons on the national and international levels.

The specific data concerning scientific production used in this analysis were obtained by assessing in the InCites’ Data Base: (1) Essential Science Indicators (ESI) composed of 22 large areas covering in brief, all the major scientific fields; (2) Web of Science (WoS) containing 251 categories of sub-areas, fields or research themes derived from the 22 scientific great areas of ESI. In both cases, data from Social Sciences and Humanities were included. The number of documents corresponding to the total of articles, conference papers and reviews published in the 5-year period 2010–2014 were extracted for further analysis. Data were obtained from the Global Comparisons Option—National Comparison Report, designed to measure the performance of the countries and institutions in selected areas of research. The search of all data were reviewed and analyzed from May to November 2015. Counting the number of publications from countries was made carefully taking in consideration the problems arising when such scores are searched (Gauffriau et al. 2007; Larsen and Von Ins 2010; Beys-da-Silva et al. 2014). These problems are related to the occurrence of double-counting of scientific literature, when comparing the production of different countries, institutions and/or fields of knowledge. This is due to the origin of each publication when it results from the cooperation between researchers connected to two or more countries, institutions or different areas. In these cases, the article will be counted more than once.

The bibliometric indicators used in this study were extracted or calculate from InCites’ Data Base as described below:

- Countries distribution: The 217 countries were divided in three groups according to their scientific contribution to the word’s total in the period 2010–2014: Group I includes the countries that have produced individually at least 1 % of the word’s articles in the period; Group II is composed of other countries each one producing from 0.1 to 0.99 % of the world sum; Group III is composed of countries with <0.1 % individual contribution to the world total.
- Number of documents: Sum of the number of documents published as full articles, conference papers and reviews of a selected area or country. These documents are relevant items published in a scientific journal covered by the Journal Citation Reports (JCR of Thomson Reuters). This study does not include Editorials, letters, news and meeting summaries, also available in the base, because usually such documents are not cited. In the text and tables we use the words articles or documents with the same meaning.
- World percent publications: Number of documents published in a subject area or country divided by the total number of documents in the world. Unless otherwise informed this indicator was obtained from the InCites’ Data Base, and it is calculated based on the number of publications without double-counting.
- Impact: Number of citations received by articles published by a country, institution, area of knowledge, or a researcher, divided by the total number of papers published in the same period. Here again the data was obtained from the InCites’ Data base. The number of citations received by an article indirectly denote the impact caused by the article over the research field and thus it is an indicator of the article’s quality.
- Impact relative to world (IRW): It concerns to the impact of an area or country relative to the world’s average impact of that area or the average of all countries together. An IRW index greater than 1.0 indicates that the impact in a specific area or country is larger than the average scientific impact of all areas together and, in the case of countries it means that the impact of a country is higher than the average of all countries.
- Double-Counting Index (DCI): This index represents the percent of double counted articles, calculated for each area or field of knowledge. In the case of the world publications it derives from the calculated sum of publications counted country by country, for which the documents published by authors from more than one country will be counted two or more times, depending on the number of foreign countries involved in the partnership. The concept was extracted from the InCites Indicators Handbook which states that “internationally collaborative document is an indicator that only takes into account if a document is international (two or more countries) or not. It does not take into account the total number of countries represented in the publication”.
- Percentage of international collaboration: The data was obtained from InCites’ Data Base; it corresponds to the percentage of articles published by authors belonging to institutions of two or more countries. Since the characterization of an article as having international collaboration depends upon the indication of authors’ addresses, we based our study exclusively on the percentage of international collaboration informed by InCites.
  The sources of other indicators used in this study are described below:
- Human Development Index (HDI): PNUD 2014 http://www.pnud.org.br/IDH/DH.aspx
- Percentage of GNP applied in R&D: 2015 World development indicators: science and technology. United Nations Educational and Cultural Organization (UNESCO) Institute for Statistics http://wdi.worldbank.org/table/5.13
- Number of scientists and engineers per million inhabitants: 2015 World development indicators: science and technology. United Nations Educational and Cultural Organization (UNESCO) Institute for Statistics
http://wdi.worldbank.org/table/5.13

## Results and discussion

### Double-counting effect in the world’ scientific production

In the period 2010–2014, the 217 countries listed in the Thomson Reuters’ InCites Data Base produced a total of 6,811,602 articles. However, considering the individual country by country contribution, the sum reaches a total of 9,064,873 articles (Table 1). The difference accounts for the double-counting effect due to publications produced in co-authorship by authors from two or more countries. Here the additional counting of 2,253,271 articles represents an increase of 33.1 % in the amount of articles listed. In this work, counted articles for each country includes the publications resulting from international collaboration. Unfortunately due to the large numbers of articles and countries in this study, fractional counting as recommended by Gaufrial and Larsen 2005, could not be applied here. As pointed above, the double-counting trait is derived from the fact that whenever a publication includes cooperation between or among researchers linked to two or more institutions, countries or fields of knowledge, the article will be counted more than once. Double-counting is inherent to the bibliometric measures of scientific production of the countries, research fields and institutions because of the steady increase of international scientific collaboration. In our study, this feature appears in the high number of articles resulting from international collaboration especially from the less productive countries together with more developed ones (see below). Thus, it is important to know its dimension because it can and in fact, it does introduce bias in the scientometric analysis of science output, especially in cases of comparisons of countries and subject areas of research. In this work we compare the data of both indicators, that is to say, with and without double-counting.

**Table 1:** World scientific production in the 22 great ESI areas: 2010–2014. The double-counting effect *Source*: InCitesTM, Thomson Reuters. Report created: 12/05/2015. Data source: Web of science

Nr.	Subject area	Number of countries	No double counting (A)	With double counting (B)	Difference (B − A) (C)	% Double counting (C/A) × 100
1	Agricultural sciences	193	190.460	240.348	49.888	26.2
2	Biology and Biochemistry	190	338.993	443.237	104.244	30.8
3	Chemistry	184	750.403	932.289	181.886	24.2
4	Clinical medicine	206	1,245,135	1,608,227	363.092	29.2
5	Computer science	148	157.999	212.953	54.954	34.8
6	Economics and business	174	121.532	166.544	45.012	37.0
7	Engineering	183	541.591	679.321	137.730	25.4
8	Environment/ecology	202	204.367	293.293	88.926	43.5
9	Geosciences	204	197.908	305.373	107.465	54.3
10	Immunology	202	118.099	174.626	56.527	47.9
11	Materials science	163	340.452	425.450	84.998	25.0
12	Mathematics	165	197.820	262.022	64.202	32.5
13	Microbiology	191	96.090	133.333	37.243	38.8
14	Molecular biology and genetics	186	205.993	299.111	93.118	45.2
15	Multidisciplinary	159	13.396	20.858	7.462	55.7
16	Neuroscience and behavior	174	239.632	323.814	84.182	35.1
17	Pharmacology and toxicology	183	179.360	228.197	48.837	27.2
18	Physics	172	551.015	820.847	269.832	49.0
19	Plant and animal science	207	335.816	463.507	127.691	38.0
20	Psychiatry/psychology	181	182.819	238.278	55.459	30.3
21	Social sciences, General	208	405.852	501.462	95.610	23.6
22	Space science	134	68.302	153.882	85.580	125.3
World		217	6,811,602	9,064,873	2,253,271	33.1

### Double-counting effect in the scientific fields

Table 1 lists the world scientific production in the 22 great areas of ESI (Thomson Reuters Data Base) in the 5 years period 2010–2014. As mentioned above, 217 countries are engaged in scientific research and publication worldwide. The question arisen from this information is: What is the contribution of each country to the production of new knowledge in each of these 22 areas and in more than 200 specific scientific fields? As shown in Table 1 all countries are developing research in the whole spectrum of scientific activities. Space Science is the area with the minimal number of publishing countries, 134, whereas Social Sciences congregated the maximal number, 208 countries, publishing in this area. As an average 182 countries are involved in publications of all scientific fields. This means that most countries, no matter how developed they are, produce scientific articles in several areas and specific fields. It is then important to know how much of such production derived from the effort of having a proper system for stimulating scientific development and what fraction represents a casual participation of the countries in these publications. In this context it is important to discriminate types or levels of international scientific cooperation, in which a country can behave as an active or passive agent. Here we show that the quantification of double-counting articles can indirectly indicate the proportion of country’s participation in the publications of a particular field. Table 1 presents both indicators: the number of articles with and without double-counting in each ESI area in the 2010–2014 period. The Double-Counting Index was calculated by subject area as described in the methods section and represents the percent ratio between the number of articles with double-counting and that of the articles without double-counting in an individual area. Publications involving 182 countries accounted 33.1 % of double-counted articles. In some areas such as Space Science for instance, the actual number of articles (68,302) generated 153,882 double-counted documents. The difference (85,580 articles) accounts for a Double-Counting Index of 125.3 % which implies that this area attracts an unusual proportion of international collaboration. Besides space science, 11 other areas (multidisciplinary, geosciences, physics, immunology, molecular biology and genetics, environment/ecology, microbiology, plant and animal science, economics & business, neuroscience & behavior and computer science) also show high level (above the table average) of double-counted articles. On the other hand the lowest values are found for Social Sciences (23.6 %), Chemistry (24.2 %), Materials Science (25.0 %) and Engineering (25.4 %).

### Double-counting effect in the scientific production of countries

As happen with the observed dispersion of the scientific production among the areas of knowledge seen in Table 1, a similar situation is detected when such distribution is considered for the 217 countries. Table 2 shows the distribution of all 217countries into three different groups, according to their contribution to the world production. Group I includes the most productive countries which individually contributed at least with 1 % for the world total. The 24 countries in Group I represent 11.1 % of the world countries but published 7,605,317 articles or 83.9 % of the total double-counted world scientific production. Group II is composed of 40 other countries and accounts for 18.4 % of the total countries, each one producing from 0.1 to 0.99 % of the world sum, thus making a total of 1,285,850 (14.2 %) of the articles’ total. Group III is composed of 153 countries (70.5 % of the total) with <0.1 % individual contribution, representing altogether 173,706 articles (1.9 %) of the world scientific output (Table 2).The Table also presents other qualitative characteristics of each country’s groups indicating the average data for: the impact, impact relative to world (IRW), percentage of cited articles, International Collaboration Index (ICI), percentage of the Country’s GNP applied in R&D and the proportion of Scientists and Engineers per million inhabitants. As shown in Table 2, several of these qualitative indicators reflected characteristics of the three groups and they will be better explored in the analysis of the country’s groups presented below.

**Table 2:** Distribuition of countries according to their individual scientific production: 2010–2014 *Source*: InCitesTM, Thomson Reuters. Report Created: 12/05/2015. Data source: Web of science

Indicators	Countries distribution			World data
	Group I	Group II	Group III	
	Countries with more than 1 %	Countries with 0.1 to 0.99 %	Countries with <0.1 %	
Number of countries	24	40	153	217
Countries % of world	11.1	18.4	70.5	100
Average HDI	0.844	0.782	0.631	0.686
Total Articles with double-counting	7,605,317	1,285,850	173.706	9,064,873
Total articles without double-counting	–	–	–	6,811,602
% World production	83.9	14.2	1.9	100
International collaboration: percentual average	43.0	55.8	85.2	60.2
% Cited articles	68.8	64.2	64.9	65.2
Average of impact factor	6.4	5.3	4.8	5.1
Impact realtive to world (IRW)	1.2	1.0	0.9	1.0
%GNP applied in R&D	2.0	1.1	0.3	2.2
Scientists and engineers per million inhabitants	3.529	2.288	510	1.268

Distribution based on the world % of scientific production of each individual country considering the double counting of articles

Table 3 lists the 24 countries belonging to Group I and presents their scientific output in the period 2010–2014, in all areas and scientific fields of knowledge. Similarly, as compared to Table 1, the countries of Group I produced most publications of the total computed for each of the 22 great areas (data not shown). Thus, in this work we named Group I as the most productive countries. Table 3 presents, besides the quantitative data of the countries, their Human Development Index (HDI). The Impact of the publications from these 24 countries varies from 9.8 (Switzerland) to 3.1 (Russia and Turkey) with an average index of 6.4 which is substantially higher than that of the world’s average (5.1).The International Collaboration Index (ICI) of countries in Group I varies from 69.1 % (Switzerland) to 20.0 % (Turkey) being the average (43.0 %) much smaller than the world’s average (60.2 %).This last value is highly influenced by the high proportion of international collaboration (85.2 %) of the countries in Group III (Table 2).Actually in Group I only four countries (Switzerland, Belgium, Denmark and Sweden)have ICI higher than the world’s average. As compared to countries with very high impact consequent to an extremely high ICI (see Table 6), countries of Group I, though having relatively high scientific impact, do not show such a strong correlation with ICI. In fact several countries of this group show the lowest ICI values among all countries. This indicates that some countries of Group I, due to their scientific leadership, possess and actively offer attractive conditions for international cooperation with other countries. Due to their sizeable scientific production, this condition does not affect significantly their ICI. On the other hand, this situation works the other way around for the countries with very small scientific production, as those of Group III. Another central aspect to be noticed is that the majority of the countries of Group I also occupy the highest level of economic and social development, what can be depicted from their Human Development Index (HDI). It is noticeable that the countries of Group I present a high average HDI (0.844), which is much higher than the world’s data. In fact, only seven countries (India, Taiwan, China, Brazil, Iran, Turkey and Russia) show index values below this level, with a variation from 0.586 (India) to 0.933 (Australia).

**Table 3:** Scientific production of countries of Group I. Quantitative and qualitative data: 2010–2014 *Source*: InCitesTM, Thomson Reuters. Report Created: 12/05/2015. Data source: Web of science

Rank	Countries	Web of Science Documents		Impact	Impact relative to world	% International collaboration^a^	HDI		% GNP applied in R&D	Scientists and engineers per million inhabitants
		Number documents	World %				Index	World rank		
1	USA	1,878,643	27.6	7.8	1.4	34.7	0.914	5	2.8	4.019
2	China	932.548	13.7	4.8	0.9	25.9	0.719	91	2.0	1.089
3	Germany	495.832	7.3	7.7	1.4	52.9	0.911	6	2.9	4.472
4	England	455.025	6.7	8.0	1.5	54.7	0.892	14	1.6	4.055
5	Japan	388.844	5.7	5.7	1.0	28.5	0.890	17	3.5	5.201
6	France	347.472	5.1	7.3	1.3	55.7	0.884	20	2.2	4.153
7	Canada	308.219	4.5	7.3	1.3	50.5	0.902	8	1.6	4.490
8	Italy	294.939	4.3	7.0	1.3	47.0	0.872	26	1.3	1.974
9	Spain	265.039	3.9	6.5	1.2	47.7	0.869	27	1.2	2.653
10	India	250.427	3.7	4.0	0.7	22.6	0.586	135	0.8	157
11	Australia	248.251	3.6	6.9	1.3	50.5	0.933	2	2.3	4.335
12	South Korea	243.989	3.6	4.9	0.9	28.8	0.891	15	4.2	6.457
13	Brazil	187.936	2.8	3.6	0.7	29.1	0.744	79	1.2	698
14	Netherlands	179.721	2.6	9.1	1.7	58.2	0.915	4	2.0	4.303
15	Russia	145.504	2.1	3.1	0.6	33.6	0.778	57	1.1	3.073
16	Taiwan	135.558	2.0	4.8	0.9	25.2	0.719	91	2.4	NI
17	Switzerland	130.691	1.9	9.8	1.8	69.1	0.917	3	3.0	4.481
18	Turkey	126.236	1.9	3.1	0.6	20.0	0.759	69	0.9	1.169
19	Iran	117.803	1.7	3.4	0.6	22.0	0.749	75	0.8	738
20	Sweden	116.155	1.7	8.1	1.5	61.0	0.898	12	3.3	6.473
21	Poland	113.011	1.7	4.2	0.8	34.1	0.834	35	0.9	1.851
22	Belgium	99.522	1.5	8.1	1.5	64.9	0.881	21	2.3	4.003
23	Denmark	73.727	1.1	8.9	1.6	61.2	0.900	10	3.1	7.265
24	Scotland	70.225	1.0	9.0	1.7	55.0	0.892	14	1.6	4.055
Total 24 Countries		7,605,317	–	–	–	–	–	–	–	81.164
Table Average		–	4.7	6.4	1.2	43.0	0.844	–	2.0	3.529
World with DC		9,064,873	–	5.1	1.0	60.2	0.686	–	–	–
World without DC		6,811,602	100	5.1	1.0	–	0.686	–	–	–
% Double counting		33.1 %	33.1 %	–	–	–	–	–	–	–

Source of % GNP and Researchers per Million people: United Nations Educational, Scientific and Cultural Organization (UNESCO) Institute for Statistics

*NI* Not informed

^a^The HDI value refers to that of the United Kingdom

Actually the countries of Group I are among the most developed ones, thus indicating the importance of science as an instrument to improve and support social and economic welfare. As mentioned by Jefrey Wadworth (2014): “Fortunately, there is ample historical evidence that research and innovation contribute, in the short and long terms, to prosperity and competitiveness, as well as to the resolution of society’s greatest challenges in areas like health, energy and security”. Unfortunately this expectation seems not to be happing with countries that can only offer some desirable advantages for cooperation, such as huge and attractive biodiversity for instance. It is also true that the countries of Group I are investing the highest proportion of their GNP in R&D and also, at the same time, possess a comfortable condition of having high number of scientists and engineers (Table 3). In fact the countries of this group apply more than 0.8 % (average 2.0 %) of their GNP in S&T and, with exception of India, they possess a high proportion (average 3529) of scientists and engineers per million inhabitants. Putting together all the quantitative and qualitative indicators, the great majority of countries in Group I constitute a kind of first league of scientific development.

Table 4 lists the 40 countries pertaining to Group II and shows their scientific output in the period 2010–2014. Together these countries produced 1,285,850 articles (14.2 % of the world’s total) also covering all areas and scientific fields of knowledge. The scientific qualitative data of Group II is summarized in Table 4. The scientific impact of the 40 countries varies from 2.8 (Nigeria) to 8.4 (Singapore) with an average index of 5.3, not too far from that of the world’s average (5.1). Their IRW average index is 1.0 (range 0.5 to 1.5), with several countries showing IRW values in the same range as those of countries of Group I. With few exceptions, most countries of this group show intermediate range of international collaboration. As shown in Table 4, the International Collaboration Index (ICI) of these countries varies from 37.0 % (Serbia) to 87.2 % (Kenya), with an average (55.8 %) smaller than the world’s average (60.2 %) but much smaller than that of Group III (85.2 %). Similarly to Group I, some countries of this group can also exert attractive influence for active international cooperation. Concerning to the Human Development Index, the countries of Group II present a HDI average of 0.782 varying from 0.504 (Nigeria) to 0.944 (Norway). Related to the percentage of GNP invested in R&D a mean index of 1.0 % was found for Group II countries. However one can see in the table that there is a great dispersion (range from 0.1 % for Indonesia to 4.2 %, Israel) of such indicator among the countries of this group. In fact about one third of these countries invest more than 1 % of their GNP in R&D. Actually these are the countries that share with others of Group I high social and economic standard of life, as also indicated by their HDI.

**Table 4:** Scientific production of countries of Group II. Quantitative and qualitative data: 2010–2014 *Source*: InCitesTM, Thomson Reuters. Report Created: 12/05/2015. Data source: web of science

Rank	Countries	Web of science documents		Impact	Impact relative to world	% International collaboration^a^	HDI		% GNP Applied in R&D	Scientists and engineers per million inhabitants
		Number documents	% World				Index	World rank		
25	Austria	67.264	0.9	8.0	1.5	66.2	0.881	21	2.8	4.704
26	Israel	64.554	0.9	7.2	1.3	49.2	0.888	19	4.2	8.282
27	Portugal	59.435	0.9	6.0	1.1	55.0	0.822	41	1.4	4.142
28	Hong Kong	57.401	0.8	6.5	1.2	66.8	0.891	15	NI	NI
29	Finland	56.887	0.8	7.8	1.4	57.8	0.879	24	3.3	7.188
30	Norway	56.608	0.8	7.2	1.3	60.0	0.944	1	1.7	5.576
31	Mexico	56.100	0.8	4.4	0.8	45.0	0.756	71	0.5	383
32	Greece	54.379	0.8	6.4	1.2	59.5	0.853	29	0.8	2.628
33	Singapore	54.350	0.8	8.4	1.5	47.9	0.901	9	2.0	6.442
34	Czech Republic	52.932	0.8	5.6	1.0	50.7	0.861	28	1.9	3.251
35	South Africa	50.563	0.7	5.2	1.0	54.2	0.658	118	0.7	405
36	Malaysia	43.988	0.6	3.5	0.7	47.5	0.773	62	1.1	1.794
37	New Zealand	42.388	0.6	6.5	1.2	58.3	0.910	7	1.3	3.701
38	Argentina	41.674	0.6	5.2	0.9	46.3	0.808	49	0.6	1.226
39	Saudi Arabia	38.196	0.6	4.4	0.8	74.9	0.836	34	0.3	NI
40	Ireland	37.708	0.6	7.8	1.4	58.2	0.899	11	1.6	3.371
41	Romania	37.258	0.5	3.6	0.7	38.0	0.785	54	0.4	945
42	Egypt	36.935	0.5	3.7	0.7	53.7	0.682	110	0.7	544
43	Chile	31.423	0.5	5.1	0.9	60.8	0.822	41	0.4	391
44	Thailand	31.383	0.5	4.8	0.9	50.2	0.722	89	0.4	543
45	Hungary	31.273	0.5	6.1	1.1	57.2	0.818	43	1.4	2.523
46	Pakistan	30.326	0.4	3.4	0.6	44.6	0.537	146	0.3	167
47	Ukraine	25.236	0.4	3.3	0.6	47.5	0.734	83	0.8	1.165
48	Serbia	24.715	0.4	3.6	0.7	37.0	0.745	77	0.7	1.381
49	Wales	24.281	0.4	8.1	1.5	51.7	0.892	14	NI	NI
50	Slovenia	19.147	0.3	5.0	0.9	49.3	0.874	25	2.6	4.217
51	Croatia	18.357	0.3	4.7	0.9	42.9	0.812	47	0.8	1.529
52	Slovakia	16.254	0.2	4.6	0.9	57.1	0.830	37	0.8	2.718
53	Colombia	16.140	0.2	5.0	0.9	62.1	0.711	98	0.2	164
54	Tunisia	14.909	0.2	3.1	0.6	54.1	0.721	90	0.7	1.393
55	Northern Ireland	11.722	0.2	7.2	1.3	57.1	0.892	14	NI	NI
56	Bulgaria	11.371	0.2	5.2	1.0	55.6	0.777	58	0.7	1.693
57	Nigeria	11.119	0.2	2.8	0.5	42.3	0.504	152	0.2	39
58	Lithuania	10.461	0.2	4.2	0.8	39.2	0.834	35	1.0	2.887
59	Algeria	9.949	0.1	2.9	0.5	59.2	0.717	93	0.1	168
60	Vietnam	9.281	0.1	4.0	0.7	78.5	0.638	121	0.2	NI
61	Estonia	8.166	0.1	7.5	1.5	60.4	0.840	33	1.7	3.339
62	Morocco	7.846	0.1	4.1	0.8	63.8	0.617	129	0.7	852
63	Indonesia	7.032	0.1	4.5	0.9	86.0	0.684	108	0.1	90
64	Kenya	6.839	0.1	6.9	1.4	87.2	0.535	147	0.8	231
Table total		1,285,850	–	–	–	–	–	–	–	80.072
Table average		–	0.5	5.3	1.0	55.8	0.782	–	1.1	2.288
World with DC		9,064,873	–	5.1	1.0	60.2	0.686	–	–	–
World without DC		6,811,602	100	5.1	1.0	–	0.686	–	–	–

Source of % GNP and Researchers per Million people: United Nations Educational, Scientific and Cultural Organization (UNESCO) Institute for Statistics

*NI* Not informed

^a^The HDI value refers to that of the United Kingdom

Besides the quantitative data presented by countries of Groups I and II, Tables 3 and 4 also show the qualitative indicators listed before. In addition, other important characteristics of the countries’ indicators related to scientific production, such as the Human Development Index (HDI), the percentage of GNP applied to R&D and the proportion of scientists and engineers related to the size of population, are indicated in the tables. Thus, taken together the qualitative data of Groups I and II could result in another kind of raking (Table 5) when the Impact index is used in order to classify the countries. Concerning specially to impact, HDI, and other qualitative indicators, the ranking of countries in Groups I and II shows several changes in positions. In fact, some countries of Group II have qualitative data similar to those in Group I, while among the most productive countries several ones do not meet the same outstanding level of this group. This confirms the lack of correlation between number of publications and impact of articles (not shown). Such differences can be seen in Table 5.

**Table 5:** Scientific production of countries of Group I and II. Ranked by impact factor: 2010–2014 *Source*: InCitesTM, Thomson Reuters. Report Created: 12/05/2015. Data source: Web of science

Rank impact factor	Countries	World Rank	Documents		Impact Factor (IF)	Impact relative to world (IRW)	% International collaboration (ICI)	HDI		% GNP applied in R&D	Scientists and engineers per million inhabitants
			Number documents	World %				Index	World rank		
1	Switzerland	17	130.691	1.9	9.8	1.9	69.1	0.917	3	3.0	4.481
2	Netherlands	14	179.721	2.6	9.1	1.8	58.2	0.915	4	2.0	4.303
3	Scotland^a^	24	70.225	1.0	9.0	1.8	55.0	0.892	14	1.6	4.055
4	Denmark	23	73.727	1.1	8.9	1.8	61.2	0.900	10	3.1	7.265
5	Singapore	33	54.350	0.8	8.4	1.6	47.9	0.901	9	2.0	6.442
6	Sweden	20	116.155	1.7	8.1	1.6	61.0	0.898	12	3.3	6.473
7	Belgium	22	99.522	1.5	8.1	1.6	64.9	0.881	21	2.3	4.003
8	Wales	49	24.281	0.4	8.1	1.6	51.7	0.892^a^	14	NI	NI
9	England^a^	4	455.025	6.7	8.0	1.6	54.7	0.892	14	1.6	4.055
10	Austria	25	67.264	0.9	7.9	1.5	66.2	0.881	21	2.8	4.704
11	USA	1	1,878,643	27.6	7.8	1.5	34.7	0.914	5	2.8	4.019
12	Germany	3	495.832	7.3	7.8	1.5	52.9	0.911	6	2.9	4.472
13	Finland	29	56.887	0.8	7.8	1.5	57.8	0.879	24	3.3	7.188
14	Ireland	40	37.708	0.6	7.8	1.5	58.2	0.899	11	1.6	3.371
15	Estonia	61	8.166	0.1	7.5	1.5	60.4	0.840	33	1.7	3.339
16	France	6	347.472	5.1	7.3	1.4	55.7	0.884	20	2.2	4.153
17	Canada	7	308.219	4.5	7.3	1.4	50.5	0.902	8	1.6	4.490
18	Israel	26	64.554	0.9	7.2	1.4	49.2	0.888	19	4.2	8.282
19	Norway	30	56.608	0.8	7.2	1.4	60.0	0.944	1	1.7	5.576
20	Northern Ireland	55	11.722	0.2	7.2	1.4	57.1	0.892^a^	14	NI	NI
21	Italy	8	294.939	4.3	7.0	1.4	47.0	0.872	26	1.3	1.974
22	Australia	11	248.251	3.6	6.9	1.3	50.5	0.933	2	2.3	4.335
23	Kenya	64	6.839	0.1	6.9	1.4	87.2	0.535	147	0.8	231
24	Spain	9	265.039	3.9	6.5	1.2	47.1	0.869	27	1.2	2.653
25	Hong Kong	28	57.401	0.8	6.5	1.3	66.8	0.891	15	NI	NI
26	New Zealand	37	42.388	0.6	6.5	1.3	58.3	0.910	7	1.3	3.701
27	Greece	32	54.379	0.8	6.4	1.3	59.5	0.853	29	0.8	2.628
28	Hungary	45	31.273	0.5	6.1	1.2	57.2	0.818	43	1.4	2.523
29	Portugal	27	59.435	0.9	6.0	1.2	55.0	0.822	41	1.4	4.142
30	Japan	5	388.844	5.7	5.7	1.1	28.5	0.890	17	3.5	5.201
31	Czech Republic	34	52.932	0.8	5.6	1.1	50.7	0.861	28	1.9	3.251
32	South Africa	35	50.563	0.7	5.2	1.0	54.2	0.658	118	0.7	405
33	Argentina	38	41.674	0.6	5.2	1.0	46.3	0.808	49	0.6	1.226
34	Bulgaria	56	11.371	0.2	5.2	1.0	55.6	0.777	58	0.7	1.693
35	Chile	43	31.423	0.5	5.1	1.0	60.8	0.822	41	0.4	391
36	Slovenia	50	19.147	0.3	5.0	1.0	49.3	0.874	25	2.6	4.217
37	Colombia	53	16.140	0.2	5.0	1.0	62.1	0.711	98	0.2	164
38	South Korea	12	243.989	3.7	4.9	0.9	28.8	0.891	15	4.2	6.457
39	China	2	932.548	13.7	4.8	0.9	25.9	0.719	91	2.0	1.089
40	Taiwan	16	135.558	2.0	4.8	0.9	25.2	0.719	91	2.4	NI
41	Thailand	44	31.383	0.5	4.8	0.9	50.2	0.722	89	0.4	543
42	Croatia	51	18.357	0.3	4.7	0.9	42.9	0.812	47	0.8	1.529
43	Slovakia	52	16.254	0.2	4.6	0.9	57.1	0.830	37	0.8	2.718
44	Indonesia	63	7.032	0.1	4.5	0.9	86.0	0.684	108	0.1	90
45	Mexico	31	56.100	0.8	4.4	0.9	45.0	0.756	71	0.5	383
46	Saudi Arabia	39	38.196	0.6	4.4	0.9	74.9	0.836	34	0.3	NI
47	Poland	21	113.011	1.7	4.2	0.8	34.1	0.834	35	0.9	1.851
48	Lithuania	58	10.461	0.2	4.2	0.8	39.2	0.834	35	1.0	2.887
49	Morocco	62	7.846	0.1	4.1	0.8	63.8	0.617	129	0.7	852
50	India	10	250.427	3.7	4.0	0.8	22.6	0.586	135	0.8	157
51	Vietnam	60	9.281	0.1	4.0	0.8	78.5	0.638	121	0.2	NI
52	Egypt	42	36.935	0.5	3.7	0.7	53.7	0.682	110	0.7	544
53	Brazil	13	187.936	2.8	3.6	0.7	29.1	0.744	79	1.2	698
54	Romania	41	37.258	0.5	3.6	0.7	38.0	0.785	54	0.4	945
55	Serbia	48	24.715	0.4	3.6	0.7	37.0	0.745	77	0.7	1.381
56	Malaysia	36	43.988	0.6	3.5	0.7	47.5	0.773	62	1.1	1.794
57	Iran	19	117.803	1.7	3.4	0.7	22.0	0.749	75	0.8	738
58	Pakistan	46	30.326	0.4	3.4	0.7	44.6	0.537	146	0.3	167
59	Ukraine	47	25.236	0.4	3.3	0.6	47.5	0.734	83	0.8	1.165
60	Russia	15	145.504	2.1	3.1	0.6	33.6	0.778	57	1.1	3.073
61	Turkey	18	126.236	1.9	3.1	0.6	20.0	0.759	69	0.9	1.169
62	Tunisia	54	14.909	0.2	3.1	0.6	54.1	0.721	90	0.7	1.393
63	Algeria	59	9.949	0.1	2.9	0.6	59.2	0.717	93	0.1	168
64	Nigeria	57	11.119	0.2	2.8	0.5	42.3	0.504	152	0.2	39

*NI* Not informed

^a^The HDI value refers to that of the United Kingdom

Group III is composed of 153 countries (70.5 % of the total) each one publishing <0.1 % of the world’s production, representing altogether 173,706 articles (1.9 %) of the world scientific output (Table 2). In this group, 74 countries (48 %) produced together in the period 2010–2014 a sum of 9009 articles, about 0.1 % of the world’s total (data not shown). In the 5 year period, 36 countries in this group published <100 articles each. On the other hand, a particular feature of Group III is the high percentage of international collaboration presented by most of its components. Among the 153 countries belonging to this group, only 15 countries had an international scientific collaboration of <65 % and none below 51 %. Therefore, an unexpected average of 85.2 % is found for the international collaboration indicator of the countries of this group (Table 2). In fact, the great majority of countries in Group III display much higher individual levels of international scientific collaboration than countries in groups I or II. As a consequence, the Impact index’s average (4.8) of Group III is very close to the world’s average (5.1) which is heavily influenced by this group of countries. Actually, as shown in Table 6, several countries of this group have the highest values of scientific Impact among all countries. Considering this distortion, one should take into account as inadequate the usual comparison of country’s impact versus the world impact.

**Table 6:** The 20 Countries with highest impact factor (above 8.0 or 1.6 fold IRW): 2010–2014 *Source*: InCitesTM, Thomson Reuters. Report Created: 12/05/2015. Data source: Web of science

Impact factor rank	Country	Group	Total articles	% World^a^	World ranking	Times cited	Impact factor (IF)	Impact relative to world (IRW)	International colaboration %
1	Vatican	III	94	0.001	179	1.198	12.7	2.5	98.9
2	Bermuda	III	171	0.003	166	1.840	10.8	2.1	91.1
3	Iceland	III	4.636	0.07	72	49.946	10.8	2.1	76.5
4	Mozambique	III	791	0.012	123	8.272	10.5	2.1	95.4
5	Republic of Georgia	III	2.640	0.04	89	27.201	10.3	2.0	74.9
6	Switzerland	I	130.691	1.9	17	1,279,879	9.8	1.9	69.1
7	Equatorial Guinea	III	20	<0.001	200	194	9.7	1.9	100
8	Gambia	III	533	0.008	137	4.952	9.3	1.8	97.2
9	Netherlands	I	179.721	2.6	14	1,626,769	9.1	1.8	58.2
10	Panama	III	1.712	0.03	98	15.520	9.1	1.8	93.4
11	Scotland	I	70.225	1.0	24	634.414	9.0	1.8	55.0
12	Denmark	I	73.727	1.1	23	652.553	8.9	1.7	61.2
13	Armenia	III	3.541	0.05	82	30.195	8.5	1.7	64.0
14	Singapore	II	54.350	0.8	33	456.868	8.4	1.6	47.9
15	Sweden	I	116.155	1.7	20	935.391	8.1	1.6	61.0
16	Belgium	I	99.522	1.5	22	808.871	8.1	1.6	64.9
17	Wales	II	24.281	0.4	49	197.130	8.1	1.6	51.7
18	Monaco	III	399	0.006	144	3.249	8.1	1.6	90.4
19	England	I	455.025	6.7	4	3,652,919	8.0	1.6	54.7
20	Austria	II	67.264	0.9	25	536.357	8.0	1.6	66.2
Total 20 countries		–	1,285,498	–	–	10,923,718	–	–	–
Table average		–	64.275	18.9	–	546.186	9.3	1.8	73.4

^a^% World WOS—without double-counted articles

Table 6 lists 20 countries for which the Impact exceeds the world’s average in values above 1.5 IRW, i.e. countries with Impact value of 8.0 or higher. As shown in the table, ten of these countries (Vatican, Bermuda, Iceland, Mozambique, Republic of Georgia, Equatorial Guinea, Gambia, Panama, Armenia and Monaco) belong to Group III (Table 2), showing IRW values from 1.6 to 2.5. Actually, this group includes the five countries with the highest Impact in the world. Another feature of these ten countries is their very small number of publications: 14,537 articles altogether or <0.2 % of the world’s total in the 5 years period, but with a very high Impact average (9.1). Additionally an extremely high international collaboration index (average of 88.2 %) is characteristic of these countries.

The distortions seen in Table 6 can be better stressed when comparing the Impact data of the countries related to their scientific production accounted area by area of the InCites Data Base. Table 7 illustrates a comparative data of the Impact values of articles published in the InCites’ 22 areas of knowledge distributed by the different countries. It shows that the same distortions seen in Table 6 are even more expressive here. The table depicts the ten highest Impact values presented by countries relative to publications in each of the 22 areas. It can be seen that rarely any country of Groups I or II occupies higher places in this chart. In contrast, among the 220 positions existing in Table 7, the great majority (168/220 or 76 % of the positions) are occupied by countries belonging to Group III. These countries show very high IF values in several areas: Multidisciplinary, Computer Science, Molecular Biology and Genetics, Physics, Space Science, Biology & Biochemistry, Engineering and Clinical Medicine. On the other hand, only eight Group I countries (Scotland, Switzerland, Netherlands, Denmark, England, USA, Canada and France) appear 25 times (11.4 %) in the chart, usually with much lower IF values. Concerning to Group II, 12 countries (Wales, Ireland, Singapore, Hong Kong, Finland, Bulgaria, Croatia, Ukraine, Colombia, Estonia, Kenya and Serbia) occupy 27 positions (12.3 %) out of the 220 possibilities.

**Table 7:** Comparative data of the IF values in the ESI’s 22 areas by countries: 2010–2014. Numbers after the country’s name indicates IF value *Source*: InCitesTM, Thomson Reuters. Report Created: 12/05/2015. Data source: Web of science. Numbers that follows the country’s names represent the IF value for that country and subject area

Areas	Top 10 IF Rank of each ESI subject area									
	1	2	3	4	5	6	7	8	9	10
Agricultural sciences	Greenland 12.3	Saint Vincent and Grenadines 11.0	El Salvador 10.5	Saint Kitts and Nevis 8.0	Fiji 7.5	Scotland 6.9	Barbados 6.8	North Korea 6.8	Panama 6.6	England 6.5
Biology and biochemistry	Suriname 41.0	Cook Islands 23.0	Netherlands Antilles 20.7	Rwanda 16.2	Bermuda 13.9	French Polynesia 13.2	Greenland 13.0	Iceland 12.9	Dominican Republic 12.7	Switzerland 11.8
Chemistry	Gambia 21.5	Nicaragua 16.2	Sierra Leone 16.0	French Polynesia 13.8	Singapore 13.0	USA 10.5	Hong Kong 10.4	Switzerland 10.3	Netherlands 10.1	Scotland 9.9
Clinical medicine	Mozambique 28.9	Botswana 27.0	Panama 22.4	Azerbaijan 22.3	Guyana 21.9	Bolivia 21.5	Zimbabwe 20.4	Equatorial Guinea 19.0	Ukraine 17.7	Haiti 16.8
Computer science	Reunion 77.9	Swaziland 34.5	Madagascar 25.0	Greenland 21.0	Jamaica 11.5	Scotland 5.7	Ethiopia 5.7	Macedonia 5.3	Iceland 5.2	Singapore 4.8
Economics and business	Madagascar 17.5	Tonga 10.0	San Marino 9.7	Burkina Faso 9.2	El Salvador 8.0	Malawi 6.5	Rwanda 6.4	W Ind Assoc St 5.5	North Korea 5.0	Kenya 4.7
Engineering	Maldives 31.0	Mauritania 11.3	Swaziland 8.0	Martinique 6.7	Wales 6.2	Antarctica 6.0	Iceland 5.7	Cote Ivoire 5.6	Denmark 5.6	Hong Kong 5.6
Environment/ecology	Dominica 16.0	Suriname 14.9	Sierra Leone 12.8	Cent Afr Republ 12.5	Guinea Bissau 12.2	Singapore 12.2	Solomon Islands 11.8	Guatemala 11.4	Switzerland 11.1	Panama 10.5
Geosciences	Comoros 24.0	Tonga 16.3	Haiti 12.4	Vatican 12.2	Seychelles 12.0	Afghanistan 11.7	Greenland 11.3	Costa Rica 11.1	New Caledonia 10.5	Eritrea 10.5
Immunology	Tuvalu 28.0	Barbados 24.5	Macedonia 19.1	El Salvador 19.0	Saint Lucia 19.0	Paraguay 16.2	Palau 15.0	Ireland 14.7	Wales 14.2	Switzerland 13.6
Materials science	French Guiana 27.0	Palau 15.0	Singapore 13.1	Madagascar 11.0	Ireland 10.4	Netherlands 9.9	Switzerland 9.6	Denmark 9.3	USA 9.3	Nicaragua 9.0
Mathematics	Cape Verde 11.5	Costa Rica 7.5	Swaziland 6.9	Jordan 4.6	Ethiopia 4.5	Turkmenistan 4.3	Guadeloupe 4.1	Dominican Republic 3.8	Serbia 3.6	New Caledonia 3.4
Microbiology	Malta 29.5	Guatemala 22.3	Monaco 20.4	Cambodia 18.7	Congo Peoples Rep 16.3	Bermuda 15.6	Liberia 15.0	Congo Democratic Republic 13.7	Tanzania 12.8	Chad 11.7
Molecular biology and genetics	Greenland 63.0	Iceland 55.9	Jamaica 30.7	Costa Rica 29.6	Rwanda 28.3	Barbados 27.6	Lithuania 25.3	Estonia 25.1	Croatia 25.0	Philippines 23.5
Multidis-ciplinary	Bermuda 207.0	Barbados 106.0	Qatar 95.8	Venezuela 80.8	Wales 79.1	Colombia 77.3	Uruguay 77.0	Croatia 62.9	Greenland 58.5	Latvia 56.4
Neuroscience and behavior	Iceland 13.47	Ireland 12.9	Wales 12.7	El Salvador 12.0	England 11.6	Scotland 11.5	Malawi 11.2	Finland 11.0	Netherlands 11.0	Dominica 10.7
Pharmacology and toxicology	Martinique 16.8	Djibouti 14.0	Liechtenstein 13.8	Iceland 12.4	French Polynesia 11.7	Dominican Republic 11.0	North Korea 10.7	Monaco 10.5	Scotland 10.2	Reunion 9.8
Physics	Bahamas 47.0	Martinique 27.0	Peru 21.1	Barbados 21.0	Republic of Georgia 18.5	Cyprus 17.7	Uganda 15.7	Ecuador 15.3	Bulgaria 14.0	Armenia 13.4
Plant and animal science	Monaco 11.2	Dominica 8.5	Bermuda 7.7	Djibouti 7.7	Netherlands Antilles 7.6	Netherlands 6.8	England 6.7	Hong Kong 6.6	Switzerland 6.6	France 6.4
Psychiatry/psychology	Madagascar 25.5	Saint Lucia 16.0	Guinea 15.0	Lebanon 12.1	Bulgaria 10.1	Trinidad and Tobago 9.7	Serbia and Montenegro 9.0	Iraq 8.4	Congo Democratic Republic 7.9	Dominica 7.7
Social sciences, general	Monaco 15.6	Equatorial Guinea 11.0	Belize 8.9	Bermuda 7.7	Nauru 7.0	Honduras 6.6	Panama 6.6	French Polynesia 6.2	Congo Peoples Rep 6.1	El Salvador 6.1
Space science	Andorra 42.0	French Polynesia 40.9	Iceland 24.6	Wales 24.6	Barbados 20.8	Denmark 18.7	Ecuador 18.7	Canada 17.6	Scotland 17.5	Ireland 16.9

## Concluding remarks

In this study, to our knowledge the first of its kind, we analyzed the effect of the unbalanced international collaboration as cause of misleading information on each country’s contribution to the scientific world output. The influence of international collaboration on the scientific production of the 217 active countries of the world for the 5 years period, 2010–2014 was analyzed. The importance of international collaboration is highly appreciated and a “Global Science Engagement” has been recently suggested by Geraldine Richmond in Science (Richmond 2016) in order to confront world challenges. Several countries including South Korea, Australia, China, Taiwan, Iran, and others, have been exploring this idea, despite they have started financing science research much later than the traditional countries. However, this encouragement towards collaboration implies the need for a more participative contribution of all partners in confronting challenges such as food quality, water disposition, healthy insurance, energy supply and security, all kinds of world problems requiring a scientific approach to be solved. In fact, as suggested by the Australians (Australian Academy of Science 2010, page 2), it must be assumed by the countries that “to meet national needs and assist national ambitious it requires increased strategic focus and commensurate support”. This applies especially to the less developed countries as seen with the BRICS, for instance (Finardi 2015).

In this work we show how International Collaboration implicates in a high percentage (ca 33 %) of double-counted world articles thus impacting several qualitative data such as citations, the scientific Impact and the Impact Relative to World (IRW). In addition we explore the possible influence of some indirect indicators such as the Human Development Index (HDI), as they relate to the scientific qualifications of the countries. The main concern raised is relative to the countries of Group III. From the analysis of the indicators presented here it can be concluded that the scientific output of the countries in Group III, represented by articles produced by themselves or in symmetric or balanced international collaboration, is highly insignificant. The great number of countries in this group (70.5 % of the whole world) is characterized by a very poor contribution to the world scientific production, though associated to an extraordinary high proportion of international collaboration, resulting in a distortive proportion of artificially qualified articles. In fact it can be assumed that most countries of Group III, as well as some ones in Group II, particularly due to the small size of their scientific community may function more passively in such collaborative effort. Collaborations in such cases appear to happen because of the existence of some unique and attractive subjects to be scientifically explored, usually restricted to some specific fields of research, very particular of these countries.

The very small number of individual articles of these countries are usually published with highly productive authors from the most developed countries, which in turn receive a great number of citations. Several authors confirmed this. As a consequence, these countries show high scientific Impact for their few publications (Table 2). Because of the large number of the countries in Group III, a distortion on the average Impact of the whole world is seen. Actually, this level of distortion is even more evident when analyzing individual areas and scientific fields (Tables 6 and 7). In contrast, Tables 3 and 4 show that 14 out of 64 countries, of which only 4 are in Group I (Switzerland, Sweden, Belgium and Denmark) and 10 others ones in Group II (Austria, Hong Kong, Saudi Arabia, Chile, Colombia, Vietnam, Estonia, Morocco, Indonesia and Kenya) show International Collaboration Index (ICI) above 60 % with mean averages for the groups of 43.0 and 55.8 %, respectively. For comparison, the great majority of countries of Group III present very high ICI, with an average of 85.2 %, thus revealing their high proportion of double-counted data. On the other hand, these countries producing very small number of articles have the greatest individual scientific Impact, resulting from intense International Collaboration, usually with the most developed countries, which implies that the articles originated from such collaboration usually receive a high number of citations. As indicated by Smith et al. 2014, in general, independently of the number of publications, high levels of international collaboration usually produce better journal placement and great number of citations and thus higher Impact. When applied to countries with very small number of publications, this trend induces a biased view of the country’s productivity and of its own qualitative contribution to the world production. In a recent review, Tahamtan et al. (2016) analyzing the factors which affect scientific citations, found that, besides other factors, “authors’ reputation, authors’ academic rank and international cooperation are stronger predictors for citations”.

Thus, concerning international attraction for cooperation, here again, the observations made by the Australians for themselves (Australian Academy of Science 2010, page 12), can be applied to the poor countries, in that they should be “more than a source of raw material or a convenient observation platform”. Such situation creates a biased view about their real scientific engagement in the effort to improve their development and to help solve the problems of their own countries. Accordingly it is observed that most of these countries present a very poor Human Development Index. Here, one relevant question concerns to the role of passive international cooperation: is it possible that the kind of non-symmetric collaboration exerted by the most developed countries, as seen in these numbers, be appropriate to help these passive countries to overcome their challenges and to create any scientific, social or economic progress? Although UNESCO (UNESCO Science Report 2010) considers the rise of international cooperation as linked to the increasing research capacity of developing countries, this assumption seems not to be the case for these countries. Inversely of what is being done, these countries should take into account that a minimal level of autonomous research is the key base for aspiring future prosperity. Several authors (Bozeman and Corley 2004; Van Raan 1998; May 1997; King 2004) suggest a similar conception.

From the above analysis it is assumed that very high levels of international collaboration, as presented by many countries, deceives the bibliometric indicators regarding the importance of their scientific production and jeopardizes their qualitative outputs. In fact these misleading data distort the analysis of the qualitative output of these countries, artificially increasing the scientific Impact of many of them, and also affecting the Impact of scientific fields of research in which they engage. Thus, it is concluded that when dealing with the qualitative contribution of countries to the world output, one must take in consideration the level of international cooperation because as seen here it can and in fact it does create false impression of the real contribution of countries to their own development.
